# Cell type-specific delivery of short interfering RNAs by dye-functionalised theranostic nanoparticles

**DOI:** 10.1038/ncomms6565

**Published:** 2014-12-03

**Authors:** Adrian T. Press, Anja Traeger, Christian Pietsch, Alexander Mosig, Michael Wagner, Mark G. Clemens, Nayla Jbeily, Nicole Koch, Michael Gottschaldt, Nicolas Bézière, Volodymyr Ermolayev, Vasilis Ntziachristos, Jürgen Popp, Michael M. Kessels, Britta Qualmann, Ulrich S. Schubert, Michael Bauer

**Affiliations:** 1 Center for Sepsis Control & Care (CSCC), Jena University Hospital, Erlanger Allee 101, 07747 Jena, Germany; 2 Jena Center for Soft Matter (JCSM), Friedrich Schiller University Jena, Phiolophenweg 7, 07743 Jena, Germany; 3 Laboratory of Organic and Macromolecular Chemistry (IOMC), Friedrich Schiller University Jena, Humboldtstrasse 10, 07743 Jena, Germany; 4 Molecular Hemostaseology, Jena University Hospital, Bachstraße 18, 07743 Jena, Germany; 5 Department of Biological Sciences and Center for Biomedical Engineering and Science, University of North Carolina at Charlotte, Charlotte, North Carolina 28223, USA; 6 Institute of Biochemistry I, Jena University Hospital, Friedrich Schiller University, 07743 Jena, Germany; 7 Chair for Biological and Medical Imaging, Technical University Munich and Helmholtz Zentrum München, Ingolstädter Landstrasse 1, 85764 Neuherberg, Germany; 8 Leibniz Institute for Photonic Technology Jena, Albert-Einstein-Strasse 9, 07745 Jena, Germany; 9 Abbe Center of Photonics, Friedrich Schiller University, Max-Wien-Platz 1, 07743 Jena, Germany

## Abstract

Efficient delivery of short interfering RNAs reflects a prerequisite for the development of RNA interference therapeutics. Here, we describe highly specific nanoparticles, based on near infrared fluorescent polymethine dye-derived targeting moieties coupled to biodegradable polymers. The fluorescent dye, even when coupled to a nanoparticle, mimics a ligand for hepatic parenchymal uptake transporters resulting in hepatobiliary clearance of approximately 95% of the dye within 45 min. Body distribution, hepatocyte uptake and excretion into bile of the dye itself, or dye-coupled nanoparticles can be tracked by intravital microscopy or even non-invasively by multispectral optoacoustic tomography. Efficacy of delivery is demonstrated *in vivo* using 3-hydroxy-3-methyl-glutaryl-CoA reductase siRNA as an active payload resulting in a reduction of plasma cholesterol levels if siRNA was formulated into dye-functionalised nanoparticles. This suggests that organ-selective uptake of a near infrared dye can be efficiently transferred to theranostic nanoparticles allowing novel possibilities for personalised silencing of disease-associated genes.

## Abstract (web summary)

A potential drug should specifically interact with its intended target in order to limit unwanted side effects. Here, the authors fabricate a biodegradable polymer nanoparticle with a fluorescent hepatic uptake transporter ligand to achieve targeted *in vivo* siRNA delivery and imaging of delivery.

## Graphical abstract

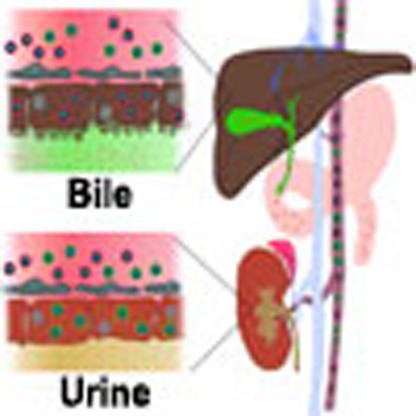

Drugs based on RNA interference (RNAi) reflect a promising class of therapeutics to interfere with virtually any protein-coding messenger RNA (mRNA), thus opening up new treatment strategies for targets that are, at present, deemed not amenable to drug development^1^,^2^,^3^,^4^. In contrast to small molecules with a wide range of different physicochemical properties, the chemical similarity of short interfering RNAs (siRNAs) permits the development of platform technologies^5^,^6^,^7^.

The most prominent obstacle in translating RNAi-based approaches into a new class of therapeutics is a specific delivery and release of siRNAs to the targeted cells, tissues and organs, particularly important when off-target effects need to be avoided^8^,^9^.

siRNAs are negatively charged, hydrophilic molecules that must overcome the hydrophobic plasma membrane^10^,^11^ to reach—in a multistep process—the RNA-induced silencing complex as their presumed site of action^12^. Cationic lipids are described as potential carriers to overcome electrostatic repulsion by binding and neutralising the negative charge of siRNAs simultaneously^13^. In addition, polycationic derivatives can effectively condense nucleic acid cargo for transfer into the cells, especially polyethylenimine (PEI), which can be considered a golden standard polymer owing to its high buffering ability for endosomal escape of siRNA to be delivered^14^. Penetrating the cell membrane may be achieved by conjugating siRNAs to small chemical moieties, such as sugar moieties, peptides or lipids^11^,^15^,^16^. These conjugation approaches enhanced the cellular entry of siRNAs, but confer organ selectivity only to a very limited extent^16^.

To fully exploit the potential of these strategies innovative delivery systems allowing active targeting are required, particularly if the systemic delivery of siRNA to internal organs is desired. Approaches previously applied to deliver siRNAs include viruses and non-viral vectors with inherent differing efficiency and toxicity^17^,^18^; however, these are far from satisfactory^19^. Depending on the application, target tissue and disease, versatile delivery strategies are of the utmost importance. Ultimately, a theranostic approach^20^,^21^ is desirable, whereby an upstream diagnostic test could account for any inter-individual variability in carrier and payload uptake such that the type and/or dose of the carrier can be individualised.

Owing to its broad metabolic repertoire, the liver, more specifically hepatocytes, constitute particularly important targets for siRNA delivery^22^,^23^. A well-characterised way of delivering siRNA cargos utilises liposomes that directly release the siRNA into the cytoplasm after fusing with the plasma or endosomal membrane^24^. This allows, albeit with limited cellular selectivity, delivery of siRNA into the liver^25^,^26^.

A higher selectivity could potentially be achieved by other uptake mechanisms, such as receptor-mediated endocytosis of polymer-based nanoparticles (NPs). Uptake transporters with organ-specific expression pattern are present in epithelial cells, for example, organic anion transporting polypeptides (OATP)^27^,^28^ found within the basolateral membrane of hepatocytes. Polymethine dyes, such as indocyanine green (ICG), which are ligands for these transporters, have been used for decades to assess hepatic excretory function^29^,^30^,^31^,^32^.

Here we report that polymethine dyes, which differ regarding their physicochemical characteristics, are eliminated with high selectivity via the hepato-biliary or renal route. These dyes can be covalently bound to polymers conferring selectivity for organ-specific uptake transporters to subsequently formed siRNA-loaded NPs. As a result, the dye-NP conjugate (DY-[NP]) reflects an escort system, for which imaging strategies to monitor uptake and clearance can be developed. This could allow the design of a platform-technology for theranostic delivery of RNAi therapeutics to the liver and, potentially, the kidney.

## Results

### NIR fluorescent dyes for functionalisation of nanoparticles

**Figure 1:**
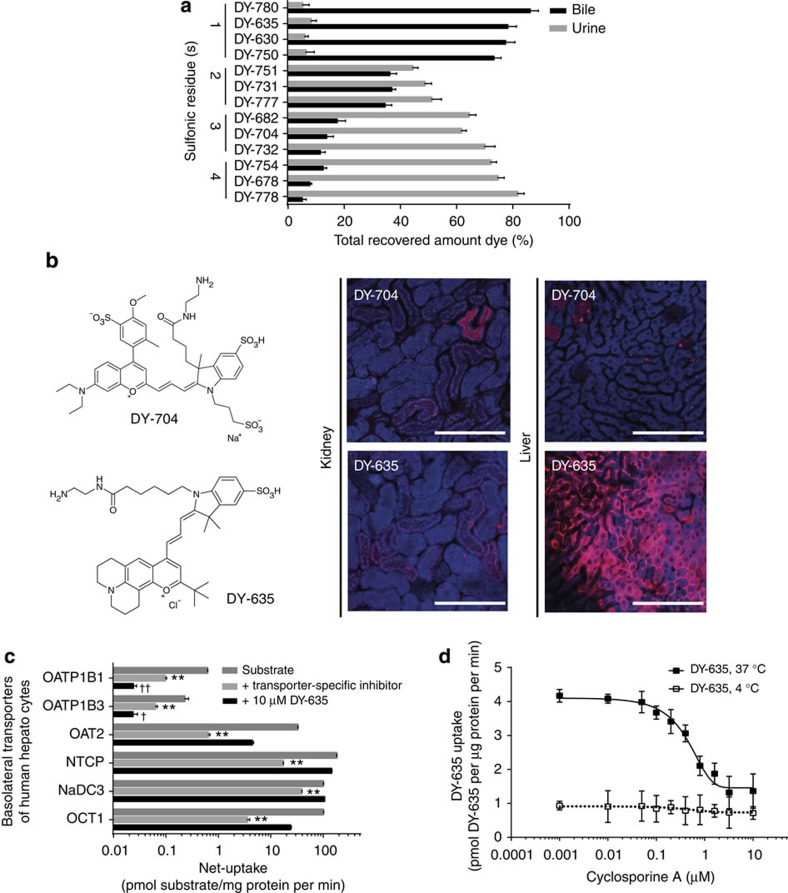
Selection of polymethine dyes with organ-selective elimination. (**a**) A variety of polymethine dyes with varying numbers of sulfonic residues were screened in rats regarding net organ-specific clearance by liver and kidneys and their applicability for biophotonic detection, for example, by IVM; (*n*=3; mean±s.e.m.). (**b**) Structures of DY-635, a dye with preferential hepatobiliary clearance and DY-704 with preferential renal elimination and their cell-specific uptake as visualised by intravital laser scanning confocal microscopy in liver and kidney (scale bars 150 μm). (**c**) DY-635 was further characterised regarding its affinity to transporters responsible for uptake of substrates into the liver in a heterologous expression system (HEK cells). While DY-635 exhibited moderate affinity to several transporters, its affinity to OATP1B1 and OATP1B3 exceeded that of rifampicin or cyclosporine A, that is, the FDA-proposed competitors for drug development purposes. Bars show mean±s.e.m.; significance was tested by one-way ANOVA followed by Tukey’s test; ** indicates *P*<0.01 for uptake of the substrate in the presence of DY-635 (hatched bars) or the FDA-proposed competitor (black bars) compared with substrate alone. † and †† indicate *P*<0.05 and *P*<0.01, respectively, for the comparison between DY-635 and the FDA-proposed competitor. (**d**) Uptake of DY-635 by primary murine hepatocytes is temperature sensitive and inhibited by cyclosporine A in a dose dependent manner with an IC_50_ of 379 nM.

We initially screened different polymethine dyes based on benzopyrylium or indolium salts with solubilising groups, that is, sulfonic residues. Although dyes containing four sulfonic residues displayed preferential renal elimination, dyes containing only one sulfonic residue were subject to hepatobiliary excretion (Fig. 1a and Supplementary Table 1). Among the studied compounds, DY-780 and DY-635 were excreted preferentially via the bile. While the optical properties of DY-780 proved to be suitable for imaging by multispectral optoacoustic tomography (MSOT), DY-635 revealed superior properties to monitor hepatobiliary clearance by intravital microscopy (IVM) (Fig. 1b). Several more hydrophilic dyes, such as DY-704 showed promising pharmacokinetic properties to target renal tubular epithelia (Fig. 1b).

To develop a theranostic delivery system for liver parenchyma, we selected DY-635 and characterised its affinity to basolateral transporters in a heterologous expression system. While DY-635 exhibited moderate affinity to several important transporters, such as the sodium-taurocholate cotransporting polypeptide (NTCP), its affinity to OATP1B1 and OATP1B3 in transfected HEK cells substantially exceeded that of the Food and Drug Administration (FDA) proposed ligand for drug development, rifampicin (ref. 33, Fig. 1c). Finally, DY-635 uptake by freshly isolated primary mouse hepatocytes was found to be rapid, temperature sensitive and inhibited dose-dependently by cyclosporine A with an IC_50_ of 379 nM (Fig. 1d).

### Polymer-synthesis and formulation of nanoparticles

**Figure 2:**
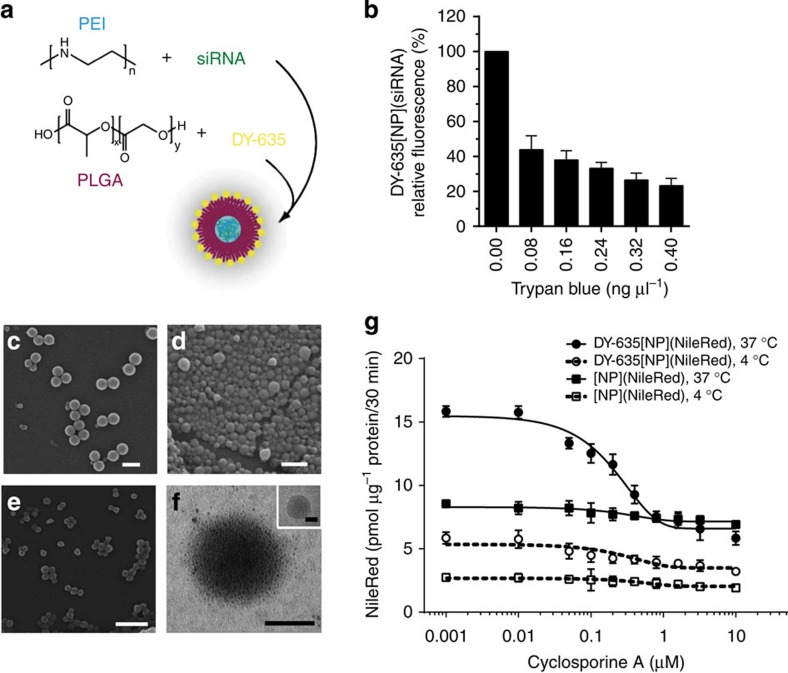
Formation and characterisation of NPs. (**a**) Dye-functionalisation of NPs. PLGA is labelled with DY-635 via EDC coupling, siRNA is complexed with LMW PEI, and NPs are formed by double emulsion procedure. (**b**) Surface fluorescence quenching of DY-635[NP](−) indicates that DY-635 is exposed at the NP surface (*n*=3, mean±s.e.m.). (**c**–**e**) Electron microscopy of NPs ([NP](−), DY-635[NP](−), DY-635[NP](siRNA) reveals regular round shape (scale bars 500 nm). (**f**) Higher power TEM of DY-635[NP](siRNA) after treatment with Cu^2+^-ions (inset shows the corresponding cryo-TEM image) suggests siRNA is encapsulated and not aggregated to the surface (scale bars 100 nm). (**g**) Uptake of DY-635 functionalised NPs is enhanced compared with non-functionalised NP and, unlike that of non-functionalised NPs is inhibited in a dose-dependent manner by cyclosporine A with an IC_50_ of 209 nM, similar to the uptake of free DY-635.

DY-635 was covalently coupled to acid terminated poly-lactic-*co*-glycolic acid (PLGA) *via* 1-ethyl-3-(3-dimethylaminopropyl)carbodiimide hydrochloride (EDC) chemistry. Covalent coupling of copolymer and dye was confirmed by size exclusion chromatography using UV/visible and refractive index (RI) detectors (Supplementary Fig. 1a) as well as UV/visible spectroscopy (Supplementary Fig. 1b). The calculated labeling efficiency for conjugation was 77%, meaning that every 130th (aimed: approximately every 100th) polymer chain bears a label. The subsequent NP formulation was accomplished by nanoprecipitation from pure PLGA (NP), PLGA with coupled dye (DY-635[NP], DY-704[NP]) and PLGA with coupled dye and loaded with additional dyes (DY-635[NP](DY-780), DY-635[NP](NileRed), DY-635[NP](ICG)). For *in vivo* gene silencing, siRNA was complexed with linear PEI of low molar mass (10 kDa; obtained by hydrolysis of poly(2-ethyl-2-oxazoline)). These complexes as well as ICG dye were encapsulated by emulsion techniques into a polymeric shell of the respective polymer (Fig. 2a). Particle surface fluorescence quenching was performed using trypan blue to demonstrate expression of the near infrared (NIR) dye on the particle surface, as this represents an essential feature for specific uptake. The investigations showed a decrease of fluorescence by nearly 75% (Fig. 2b). This indicates strongly that the dye is exposed to the particle surface and supports the notion that the dye moiety is responsible for targeting properties. The formed NPs were further characterised in detail by different methods to investigate their size, shape and surface charge (Table 1). The siRNA was encapsulated achieving an efficiency of 87.4±2.5% (Table 1) as determined by photometric phosphate quantification. Scanning electron microscopy (SEM) measurements (Fig. 2c–e) showed no significant differences between [NP], DY-635[NP](−) and DY-635[NP](siRNA). The zeta potentials, representing the surface charges of the NPs, are shown in Fig. 2c, indicating negative charge for pure PLGA, DY-635[NP]s and loaded DY-635[NP]s (Table 1).

DY-635[NP](siRNA) were investigated in further detail using asymmetric flow field-flow fractionation (AF4). A monomodal and nearly monodisperse distribution with low particle polydispersity index (1.09) (Supplementary Fig. 1c), a radius of gyration (*R*_g_) of 70 nm and a hydrodynamic radius (*R*_H_) of 90 nm, which is in accordance to batch dynamic light scattering (DLS) measurements, could be determined. The shape ratio (*R*_g_/*R*_H_) of 0.78 obtained by AF4 measurements (Supplementary Fig. 1d) corresponds to the theoretical value of a hard sphere (0.775)^34^. To confirm that the siRNA-PEI complexes are encapsulated inside the NPs and not aggregated onto their surface, individualised transmission electron microscopy (TEM) (Fig. 2f) as well as cryo-TEM measurements (inset Fig. 2f) were performed. The PEI core of DY-635[NP](siRNA) was counterstained with Cu^2+^ ions, demonstrating that the siRNA-PEI complexes were located inside the NPs. Furthermore, bio- and haemo-compatibility were studied showing neither haemolysis nor aggregation of erythrocytes (data not shown), supporting the successful encapsulation of the siRNA-PEI complexes. We then tested whether DY-635 conjugated to NPs would retain selective uptake by primary hepatocytes. Similar to what was observed with the free dye (Fig. 1d) DY-635[NP](−) were rapidly accumulated in a temperature sensitive manner by primary hepatocytes and dose-dependently inhibited by cyclosporine A with an IC_50_ similar to that observed for inhibition of uptake of the free dye (209 nM, Fig. 2g).

### Body-distribution and traceability of DY-635[NP]

**Figure 3:**
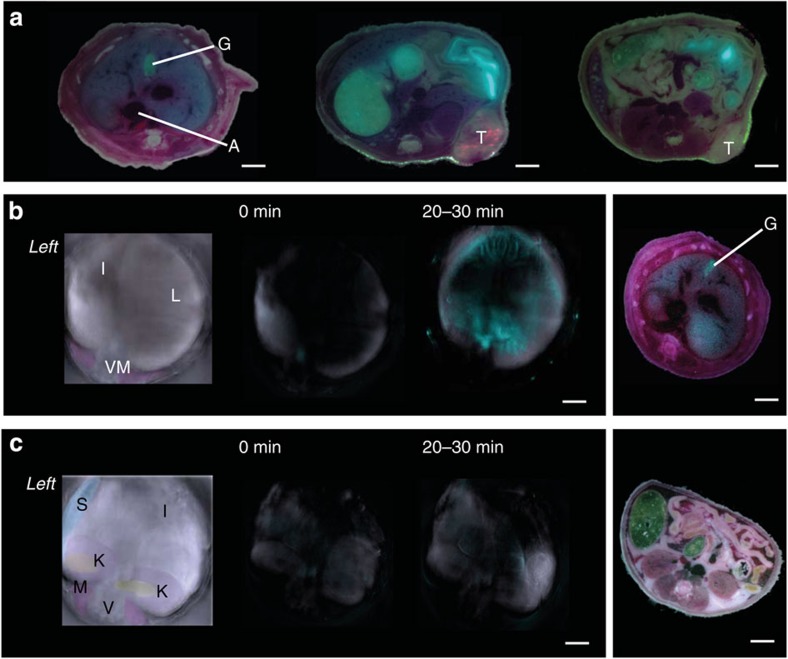
Biodistribution of NPs. (**a**) Body distribution of dye-functionalised NPs with an infrared cargo (DY-635[NP](DY-780)) in an athymic Nude-Foxn1^nu^ mouse bearing a MDA-MB-231 human breast cancer xenograft (‘T’) confirmed selective uptake and excretion via the hepatobiliary route as reflected in accumulation of the dye in the gall bladder (‘G’). (**b**,**c**) CD1 mice injected with the same NP and assessed by multispectral optoacoustic tomography acquiring image data from several wavelengths (690, 710, 750, 770, 780, 800, 810 and 850 nm, 4 frames/wavelength, 0.1 mm step size) over an abdominal area of 1.7 cm using a frequency of 54.55 kHz (scale bars 5 mm). (**b**) Sections through the upper abdomen are single-spectral that elucidate anatomical structures (left panel; ‘I’ intestines, ‘L’ liver, ‘V’ vertebral column, ‘M’ autochthonous back muscles and ‘A’ aorta) or processed multispectrally to visualise the dye cargo (DY-780) immediately prior to injection (0 min) or during early uptake (20–30 min after injection). Far right panel: Corresponding cryosection of the upper abdomen; ‘G’ indicates gall bladder containing the dye. (**c**) Subsequent sections through the lower abdomen processed for structural information (left panel; in addition the kidneys (‘K’) and spleen (‘S’) are visible) middle and right panels are processed to visualise the dye cargo. The IR dye is restricted to the upper abdomen and depicts liver and biliary tree.

**Figure 4:**
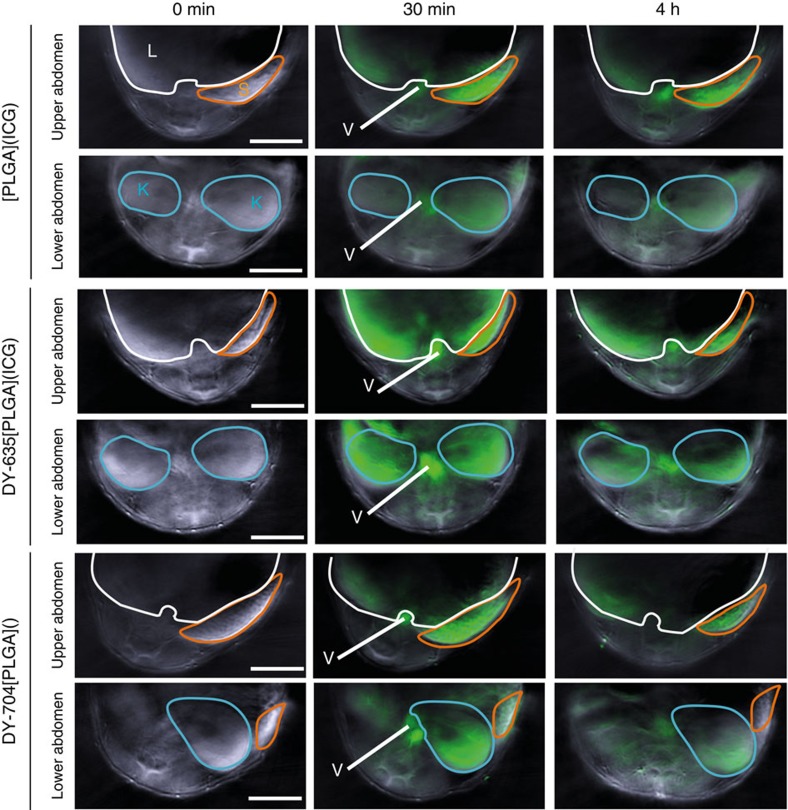
Organ distribution of dye-functionalised NP. MSOT images of non-functionalised NP or NP functionalised with DY-635 with ICG as cargo or functionalised with DY-704. The dye-moiety (in case of DY-704) or ICG cargo (in the case of DY-635 or non-functionalised NP) were used for imaging by MSOT; NP are shown in green irrespective of the dye moiety. All NP were identified in the circulation (V=retroperitoneal vessels) and showed uptake by spleen (S) indicating phagocytic clearance; however, only DY-635 functionalised NP showed significant uptake by the liver (L), whereas DY-704 functionalised NP had an increased accumulation in the kidneys (K) (scale bars 5 mm)

**Figure 5:**
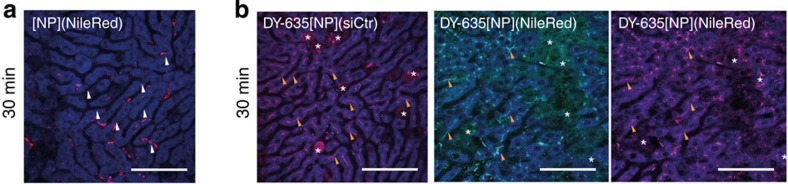
Cell-type distribution of dye-functionalised NP. (**a**) *In vivo* laser scanning confocal images of mouse liver (blue) 30 min following injection of bare PLGA-NPs containing nile red (red). These appear almost exclusively in sinusoidal lining cells (Kupffer cells, white arrow; scale bar 100 μm), (**b**) NP functionalised with DY-635, in contrast, accumulate in hepatocytes where the NileRed cargo (green) and DY-635 (red) are found either in bile canaliculi (orange arrows) or in hepatocyte cytoplasm (*) (scale bars 100 μm).

Having demonstrated that organ specificity can be transferred to dye-functionalised NPs, body distribution of DY-635[NP], carrying contrast agents as cargo, was analysed regarding active versus passive targeting properties. Athymic Nude-Foxn1^nu^ mice bearing a highly vascularised MDA-MB-231 human breast cancer xenograft were injected with DY-635[PLGA](DY-780) and analysed after cryoslicing by low-power fluorescence microscopy. Although liver, gall bladder and subsequently the intestinal lumen showed dye signal suggesting uptake by hepatocytes followed by biliary excretion, no dye signal was detected in the tumour (Fig. 3a). This is indicative of primarily active targeting to liver parenchyma rather than passive targeting even in the presence of a highly vascularised tumour. Subsequently we tested whether the optical properties of DY-780 would allow non-invasive imaging of organ distribution using MSOT in anaesthetised, healthy CD1 mice. Consistent with the uptake pattern in nude mice, a DY-780-derived signal in liver parenchyma, biliary tree and gut could be detected by MSOT indicating that hepatocytes take up and secrete DY-780 into bile (Fig. 3b,c). Further experiments were conducted to confirm the need for a hepatoselective dye to target liver parenchyma. MSOT confirmed unspecific uptake of all applied NPs in spleen and to a much lesser extent in the liver (Fig. 4a). While NPs with DY-635 as targeting moiety were extensively accumulated by the liver, either absence of dye or functionalising with DY-704, that is, a dye that is not a substrate for hepatocyte transporters, failed to show active targeting to the liver. Moreover, *in vivo* laser scanning microscopy indicated that bare NPs carrying nile red were taken up by the liver and found almost exclusively in sinusoidal lining cells (primarily Kupffer cells). In contrast, DY-635 functionalised NPs were taken up by hepatocytes with the nile red cargo retained in the cytoplasm and the DY-635 transported into the bile (Fig. 5a,b). Thus, the NPs maintain their affinity to hepatic uptake transporters and allow tracking of drug delivery by biophotonic strategies.

### Uptake mechanisms of DY-635 and DY-635[NP](−)

**Figure 6:**
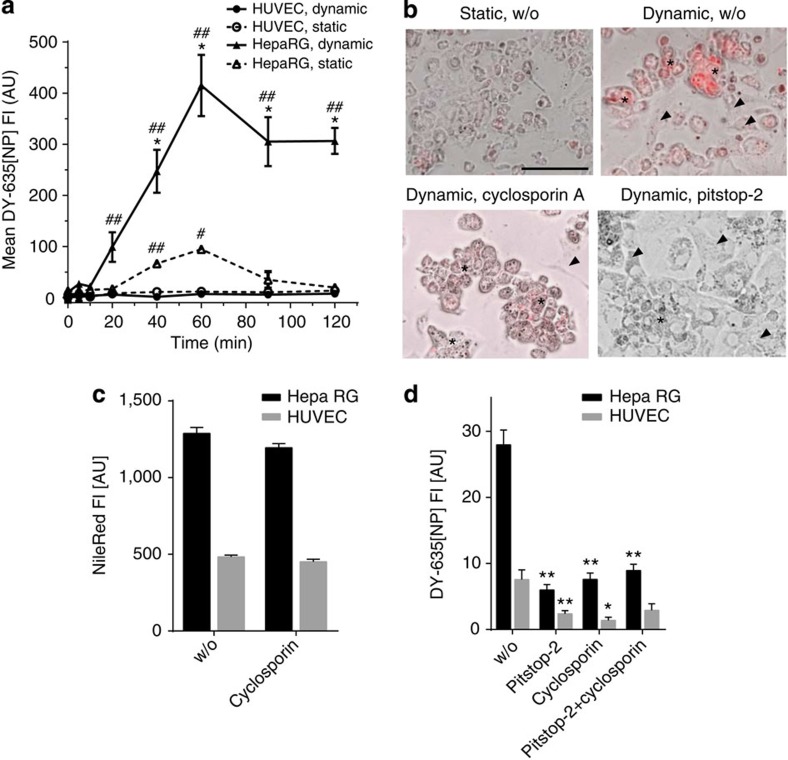
Uptake of NPs exposing DY-635 on their surface *in vitro*. (**a**) A microfluidic-assisted ‘organoid’ composed of co-cultured HepaRG and HUVEC demonstrates an increased uptake by the hepatocytes preferentially if cells are subjected to flow conditions (‘dynamic’). Under flow conditions, this organoid better recapitulates the function of the liver *in vivo* by circulating fluid containing NP through the equivalent of the space of Disse. (**b**) Representative overlay of brightfield and fluorescence images of HepaRG cells differentiated to hepatocytes (stars) or endothelia like (arrow head) cells cultured under static or dynamic conditions reflect increased uptake of DY-635[NP](−) (red) after 90 min of incubation if cells are subjected to flow. This uptake can be inhibited by cyclosporine A as well as Pitstop-2. DY-635[NP](−) uptake was assessed by epi-fluorescence microscopy (Cy5 channel); (scale bar 100 μm). To characterise cell selectivity and uptake mechanisms, further experiments were conducted. (**c**) Uptake of DY-635[NP](−) is inhibitable by cyclosporine A, a ligand for OATPs and NTCP in HepaRG but not in HUVEC that do not express these transporters; inhibition by Pitstop-2 suggests clathrin-mediated endocytosis as molecular mechanism of cellular uptake. (**d**) Cyclosporine A failed to affect the overall less pronounced basal uptake of [NP](nile red) that are not exposing DY-635 on their surface. For the statistical analysis a generalised mixed model was applied, taking into account dependent (time, flow condition) and independent (cell type) data. *Post hoc* analysis was performed using Tukey’s test. In panel (**a**) * indicates *P*<0.05 comparing respective static versus dynamic conditions; # indicates *P*<0.05, ## indicates *P*<0.01 for the comparison between HepaRG and HUVEC. In panel (**d**), * and ** indicate *P*<0.01 and *P*<0.01 respectively, for the uptake of DY-635[NP](−) in HepaRG or HUVEC in the absence of a co inhibitor (w/o) compared with the uptake in presence of cyclosporine A or Pitstop-2.

A microfluidic-assisted organoid that mimics the sinusoidal anatomy enabling us to study uptake kinetics of a human hepatocellular cell line (HepaRG) in the presence of human umbilical vascular endothelial cells (HUVEC) under static or flow conditions was used to assess mechanisms of uptake (Supplementary Fig. 2). As shown in Fig. 6a,b, uptake of DY-635[NP](−) was significantly increased under conditions of flow compared with the static culture. In the presence of flow, the pressure gradient in the flow chamber results in efflux of perfusate (including NPs) out of the ‘vascular’ space into the space surrounding the HepaRG cells (analogue of space of Disse *in vivo*) at the inflow and back into the vascular space at the outflow. In this way, DY-635[NP] are delivered to the HepaRG cells similar to what occurs in the liver *in vivo*. In a co-culture of HepaRG and HUVEC cells we could also demonstrate selectivity of DY-635[NP](−) to hepatocytes and inhibition of NP uptake by cyclosporine A and Pitstop-2 supporting an OATP-dependent clathrin-mediated endocytosis (Fig. 6b,c). Basal uptake of NP made from unfunctionalised PLGA and loaded with NileRed for detection was not affected by cyclosporine A (Fig. 6d).

**Figure 7:**
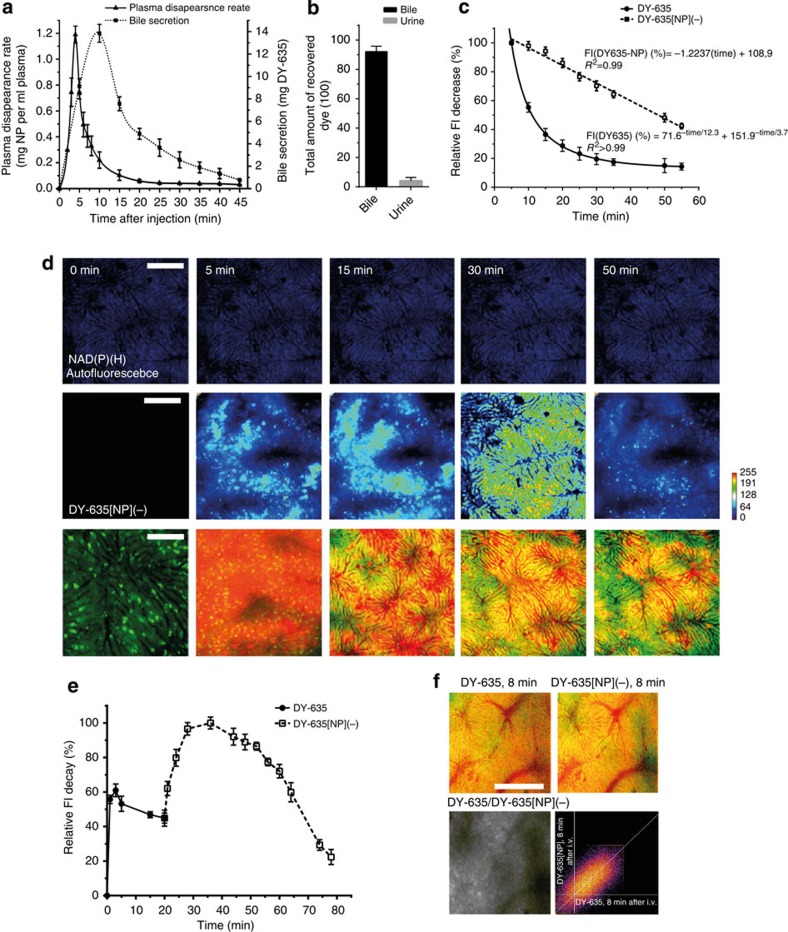
Pharmacokinetics of DY-635[NP](−). (**a**) Time course of arterial plasma concentration after central venous application of DY-635[NP](−) (left ordinate) at time ‘0’ and appearance of the desorbed dye in bile (right ordinate) (*n*=3, mean±s.e.m). (**b**) Percentage of recovered free dye in bile and urine after administration of DY-635[NP](−). (**c**) Fluorescence decay over liver parenchyma in the Cy5 channel after administration of unbound DY-635 or DY-635[NP](−) revealing exponential decay for the free dye and almost linear decay for DY-635[NP](−)-associated fluorescence (*n*=3 per 25 ROIs per n; mean±s.e.m.). (**d**) Intravital epifluorescence microscopy to visualise acinar distribution of DY-635[NP](−). Upper panel background fluorescence of the liver (blue). Middle panel: Heatmap (blue: low intensity, red: high intensity) reflecting signal distribution which is associated with the pericentral region of the liver lobule early upon injection of DY-635[NP](−) and spreads towards midzonal and even periportal region over time. Lower panel: corresponding false colour images of association of DY-635-associated fluorescence (red) with liver parenchyma (green): fluorescence is restricted to the vascular compartment early upon injection (5 min); yellow signal indicating colocalisation of dye with liver parenchyma is increasingly observed over time. (scale bars 100 μm). (**e**) Theranostic use of the free dye DY-635 to predict subsequent uptake of DY-635[NP](−). DY-635 was injected at time ‘0’ followed by injection of DY-635[NP](−) at 20 min. (**f**) Intensity of fluorescence signal over liver parenchyma; right panel: signals obtained upon injection of free dye and dye-functionalised nanoparticles are correlated. (*n*=2, 20 ROIs per n; mean±s.e.m.) (scale bar 100 μm).

Since high selectivity for hepatocytes was confirmed for DY-635[NP](−) and because liver parenchyma could secrete free DY-635 into bile^32^, we performed *in vivo* pharmacokinetic measurements of DY-635[NP](−) to assess (1) plasma disappearance, (2) hepatocellular clearance and (3) appearance of the dye in collected bile. After central venous administration of DY-635[NP](−), plasma kinetics in arterial blood revealed a peak at 4 min after administration. Disappearance followed an exponential decay with biliary excretion reaching its maximum at 15 min (Fig. 7a). Monitoring of parallel urine samples was consistent with almost exclusive secretion of DY-635 from DY-635[NP](−) *via* the hepatobiliary route. When biliary recovery of DY-635 was quantified, the ratio of secreted DY-635 to injected DY-635 bound to DY-635[NP](−) exceeded 90% after 45 min (Fig. 7b). A change in the relative fluorescence intensity decrease in the hepatocyte from an exponential decay (free dye) to an almost linear kinetic after injection of DY-635[NP](−) was observed supporting the assumption that DY-635 and DY-635[NP] are processed differently and that rate limiting intracellular reactions have to occur prior to secretion of the dye (Fig. 7c). Sublobular distribution of DY-635[NP](−) with liver tissue, as shown in Fig. 7d, confirmed the highest concentrations around central veins with a steady decrease towards the lobular periphery early upon injection (Fig. 7d, upper and middle panel). The fluorescence signal subsequently displayed a spreading towards the periportal region (Fig. 7d, lower panel).

To assess the potential use of the free dye to predict uptake of DY-635[NP] in a theranostic system, we injected DY-635 and DY-635[NP](−) sequentially. After DY-635 injection an increase in fluorescence intensity was observed followed by an exponential decay. Administration of DY-635[NP](−) 20 min after DY-635 injection resulted in a second peak with a linear decay as shown for the individual compounds (Fig. 7e), whereas distribution of DY-635 and DY-635[NP] were similar (Fig. 7f)

### Cell type-specific RNAi of *Hmgcr* using DY-635[NP](siHMGCR)

**Figure 8:**
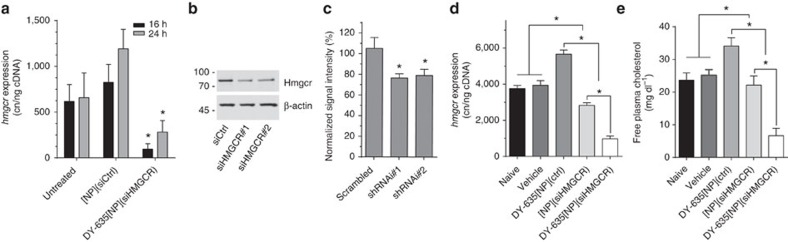
*hmgcr* RNAi using DY-635[NP] as carrier. (**a**) A significant reduction in *hmgcr* gene-expression was achieved with a maximum at 16 h after transfection in naive Hepa1-6 cells. In contrast to DY-635[NP](siRNA) an increase in *hmgcr* was observed after application of the carrier (DY-635[NP](siCtrl)). Mean±s.e.m. *P<0.05 compared with respective siCtrl by ANOVA and Tukey *post hoc*. (**b**,**c**) To validate siRNAs, the efficacy of two RNAi sequences (shRNA#1, shRNA#2) against *hmgcr* was analysed in a heterologous expression model using western blot (**b**), and addressed by determining endogenous HMGCR levels in HepG2 cells using anti-HMGCR immuno-fluorescence analysis (**c**). A significant reduction in hmgcr protein could be achieved with both RNAis; for further RNAi experiments RNAi sequence#2 was selected. Mean±s.e.m. *P<0.05 versus control by ANOVA and Dunnett’s test. (**d**) *In vivo* RNAi results in mice injected with [NP](siHMGCR) (*n*=9) or DY-635[NP] loaded with siHMGCR (*n*=12) or a control RNA (siCtrl) (*n*=12); animals injected with the vehicle (5% sterile glucose solution; *n*=4) served as sham controls. *hmgcr* gene expression was analysed using relative quantification compared with untreated anminals (*n*=4) in RNA prepared from liver tissue by RT-qPCR. Consistent with the results of the cell culture, DY-635[NP](siCtrl) induced the *hmgcr* gene expression while [NP](siHMGCR) blunted this effect but failed to lower the *hmgcr* expression compared with untreated or sham animals. Using similar siHMGCR amounts encapsulated in DY-635[NP](siHMGCR), *hmgcr* expression was lowered by 75% compared with untreated animals. Mean±s.e.m. *P<0.05 by ANOVA and Tukey test. (**e**) Altered gene expression of *hmgcr* was reflected in plasma-cholesterol levels of these animals. Mean±s.e.m. by ANOVA and Tukey test.

Next, we performed functional analysis of DY-635[NP](siRNA) delivery of siRNA in Hepa1-6 cells. In comparison to untreated controls, we observed a decrease in *hmgcr* mRNA-levels of 75% after 16 h and of 60% after 24 h. In contrast, DY-635[NP](siCtrl) loaded with a control siRNA showed some stimulatory effect on HMGCR mRNA at 16 and 24 h after treatment so that the total (net) inhibitory effect of *hmgcr* RNAi was even more substantial in comparison to DY-635[NP](siCtrl) at both 16 and 24 h (Fig. 8a).

As successful RNAi was critical to our therapeutic strategy, we next validated the RNAi effects directly at the protein level. We first demonstrated the knockdown of the catalytic domain of HMGCR fused to green fluorescent protein (GFP) and expressed under a cytomegalovirus-promotor from vectors that additionally drive either specific RNAi or scrambled RNAi in HEK293 cells using quantitative immunoblotting (Fig. 8b).

Second, we directly assessed the knockdown of endogenous HMGCR by quantitative immunofluorescence analyses in HepG2 cells. Both RNAi constructs led to a significant reduction of anti-HMGCR immunosignals (Fig. 8c; Supplementary Fig. 3). We then tested whether knockdown with DY-635[NP](siHMGCR) would be effective *in vivo* using a mouse model. After two injections via a venous port, liver tissue was analysed. An absolute decrease of *hmgcr* mRNA of 70 % versus untreated animals was achieved (Fig. 8d). This decrease in mRNA was functionally significant, as DY-635[NP](siHMGCR) treatment-mediated decrease of *hmgcr* mRNA resulted in a significant reduction of plasma cholesterol compared with the controls (Fig. 8e). Again it was observed that treatment with DY-635[NP](siCtrl) lead to an up-regulation of *hmgcr* accompanied by an increase in *hmgcr* mRNA (7d) and plasma-cholesterol levels (Fig. 8e). This is likely the result of low level activation of lipid metabolic pathways known to be associated with transient penetrations of the cell wall^35^.

## Discussion

In this study, we characterised multifunctional NPs generated by covalent conjugation of a fluorescent dye with known hepatobiliary clearance to a biodegradable polymer (PLGA) as a carrier system for PEI-complexed RNAi therapeutics^36^,^37^,^38^,^39^. The highly organ-selective uptake of the NIR dye was maintained after covalent binding to PLGA-NPs. Furthermore, the NIR dye moiety allowed monitoring of distribution, uptake and clearance using biophotonic approaches. Assessing the pharmacokinetics of DY-635[NP](−) confirmed hepatobiliary clearance exceeding 90% in <1 h. The conjugate thus represents a fundamentally new class of theranostic delivery systems with a highly efficient active-targeted delivery and the option, based on the preceding injection of the free dye, to predict the tissues that will eventually take up a nanoformulated drug before it is administered. Since these dyes can be modified with regards to their physicochemical properties (as shown exemplarily for DY-704 which is eliminated primarily via the kidney), a platform technology to deliver dye-functionalised NPs to a variety of cells and tissues expressing these transporter systems seems feasible. We could identify organic anion transporter proteins as the transporters responsible for uptake of DY-635. Their critical role in the active targeting of DY-635[NP](−) to achieve basolateral uptake into hepatocytes is supported by the observation of cyclosporine A-sensitive uptake by HepaRG cells cultured under flow conditions. Our *in vitro* and *vivo* findings suggest a model where the DY-635[NP]s are immobilised at the cell surface of hepatocytes through interaction of DY-635 with OATPs followed by subsequent clathrin-mediated endocytosis. Because of their size (170 nm), DY-635[NP](−) cannot be transported directly by the OATPs^40^ and endocytosis after immobilisation via binding to the uptake transporters on the basolateral membrane is the likely mechanism. Dahlman *et al.*^41^ have recently reported siRNA encapsulated in NPs that specifically target vascular endothelial cells. The NP appear to be taken up via receptor mediated endocytosis, but this mechanism is distinct from the dye functionalised NPs that we describe. The vascular endothelium is in direct contact with the intravascular space and exhibits effective but not very specific endocytosis. In contrast, hepatocytes are separated from the vascular space and our technology exploits recognition of the transporters that are very specific to the hepatocytes. Thus while the technology of Dahlman *et al.* appears to be very effective for targeting vascular lining cells, it is not likely to be able to target nonvascular cells.

Likewise, DY-635 labelled PLGA cannot be secreted directly into bile due to its molar mass (MW) of 12 kDa (non-modified polymer) and hydrophobicity. Based on the data in Fig. 5a, showing hepatocyte uptake and excretion of both, nile red cargo and DY-635 as functionalising moiety, degradation by intracellular esterases of PLGA with desorption of DY-635 and subsequent secretion into bile is likely. This is also supported by the observation that the decay of fluorescence followed an exponential function for the free dye, while it was delayed and almost linear for the DY-635[NP](−)-associated signal. It would also be consistent with a multistep processing within the endosome, with disruption and release of the payload into the cytosol, supported by the cationic polymer PEI^42^,^43^. Escape of siRNA from the endosome is a critical and rate-limiting step for delivery approaches^44^. Quantification of this delivery for lipid-based NP (LNP) delivery has been previously determined to be in the range of 1–2% of the applied siRNA^44^. Thus, confirmation of uptake of NPs does not necessarily imply functional delivery of the RNAi therapeutic. Although assessment of the intracellular handling of the cargo is beyond the scope of the present study, we demonstrated a significant inhibition of cholesterol biosynthesis with encapsulation of a siRNA directed against *hmgcr* into DY-635[NP]. *Hmgcr* was chosen as an exemplary pathway that reflects a key metabolic function of the liver but also based on the observation that DY-635[NP](−) increased plasma cholesterol while toxicology screening otherwise revealed no unwarranted side effects. The observed increase in *hmgcr* and plasma cholesterol with the empty carrier is a phenomenon likely to be associated with the penetration of the cell membrane NPs leading to an adaptive increase of cholesterol biosynthesis as described for endocytosis^45^ or in response to pore-forming toxins^35^. This side effect may confer protection in the acute setting^46^ but is of concern if repeated administration seems required. The increase in plasma cholesterol could be blunted if siRNA directed against *hmgcr* was the cargo in non-functionalised NPs, or substantially decreased if the same NPs were functionalised by DY-635 to target hepatocytes as a primary site of cholesterol synthesis.

During degradation of DY-635[NP](siRNA) and the associated release of the siRNA into the cytosol, DY-635 is presumably desorbed from the polymer and secreted into bile. The zonal distribution of the dye during this process with primary uptake in the pericentral region and subsequent cell-to-cell transport to midzonal and periportal regions of the low-molar mass dye (potentially also an RNAi therapeutic) is likely to explain the spreading of the fluorescence signal ostensibly through gap junctions^47^,^48^.

IVM also confirmed that uptake of DY-635[NP](−) was almost exclusively associated with hepatocytes, primarily in zone 3 of the acinus. No significant accumulation in non-parenchymal cells, most notably Kupffer cells that are primarily sited in zone 1 of the acinus and that take up significant portions of LNPs^49^, was observed for DY-635[NP](−). This extends the concept of active targeting of the liver (with a high proportion of phagocytic and immunocompetent cells) to an active and highly specific targeting of liver parenchymal cells, that is, the primary goal for targeted interventions into metabolic pathways.

While fluorescence microscopy is a suitable tool to study these fundamental questions regarding uptake and fate of DY-635[NP](−), the ability to monitor body distribution non-invasively by MSOT provides new opportunities to guide the delivery of RNAi therapeutics. Theranostics, a linguistic blend of diagnostics and therapy, reflects an increasingly important concept in personalising health care. This aims to explain inter-individual and population-to-population variabilities in health intervention outcomes using diagnostic tests such that either the type or extent of the intervention can be individualised^20^. Our present approach allows this concept to be applied to the delivery of nano-formulated active drugs, in particular those with significant off-target effects, by predicting the targeted cells and tissues individually before administration of the carrier and cargo.

In summary, the present study demonstrates that uptake receptors can be targeted very efficiently with polymethine dyes. Using DY-635, a dye with known hepatobiliary clearance, we demonstrate that this approach extends the concept of delivery to the liver, for example, with LNPs to exclusive targeting of hepatic parenchymal cells. In addition to exploring a novel, well-defined uptake route the use of dyes to functionalise the NPs also enables their tracking, even non-invasively by MSOT^50^,^51^,^52^. This suggests that organ-selective uptake of a dye can be transferred to NPs to generate theranostic drug carriers allowing fundamental novel options in personalised health care.

## Methods

### Animals

Animal studies were conducted in accordance with animal welfare legislation under pathogen-free conditions in the animal facility of the Jena University Hospital or the Helmholtz-Center Munich. During all procedures and imaging methods, animals remained under deep general anaesthesia using Isoflurane and pain-reflexes were assessed to gauge the depth of anaesthesia.

### *In vivo* pharmacokinetics of various polymethine dyes

Catheters were placed in the jugular vein, carotid artery, bladder and bile duct of male Wistar rats (300–400 g body weight (BW)). After i.v. administration of DY-635 (13 pmol per g BW), DY-630 (13 pmol per g BW), DY-750 (13 pmol per gBW), DY-751 (13 pmol per g BW), DY-731 (13 pmol per g BW), DY-777 (14 pmol per g BW), DY-682 (13 pmol per g BW), DY-704 (17 pmol per g BW), DY-732 (13 pmol per g BW), DY-754 (17 pmol per g BW), DY-678 (14 pmol per g BW), DY-778 (14 pmol per g BW) or 6.5 μg DY625-[NP] per g BW, bile was collected every 5 min and 200 μl arterial blood taken at defined times into heparinised tubes. DY-635 fluorescence in bile and plasma was measured in a spectrofluorometer (FluoStar Optima, BMG Labtech). Results are mean±s.e.m. of three biological replicates.

### Analysis of DY-635 interaction with human hepatic basolateral transporters

HEK293 cells were transfected with OATP1B1 (*SLCO1B1*, NM_006446), OATP1B3 (*SLCO1B3*, NM_019844), OCT1 (*SLC22A1*, NM_003057.2), OAT2 (*SLC22A7*, NM_006672), NTCP (*SLC10A1*, NM_003049), NaDC3 (*SLC13A3*, AF154121) or the empty vector (pcDNA5).Transfected HEK293 cells were seeded at 2 × 10^5^ cells per well and cultured for 3 days. To determine the interaction of DY-635 with different transporters mock- and transporter-transfected cells were incubated with radio-labelled substrates for OATP1B1 ([^3^H]estrone-sulfate, 30 nM), OATP1B3 ([^3^H]sulfobromphthalein, 50 nM), OCT1 ([^3^H] 1-methyl-4-phenylpyridinium iodide, 10 μM), OAT2 ([^3^H]cGMP, 10 μM), NTCP ([^3^H]estrone-sulfate, 10 μM) or NaDC3 ([^14^C]succinate, 10 μM), in the absence and presence of 10 μM DY-635 or an inhibitor for OATP1B1 (rifampicin, 20 μM), OATP1B3 (rifampicin, 20 μM), OCT1 (decynium22, 50 μM), OAT2 (indomethacin, 100 μM), NTCP (cyclosporine A, 50 μM) and NaDC3 (succinate, 100 μM)

### Hepatocyte isolation

Male FVB/NRj were sacrificed using cervical dislocation, The abdomen was opened and the portal vein catheterised and perfused briefly with pre-warmed Dulbecco’s PBS (Sigma Aldrich) containing 0.5 mM EGTA and 0.1 % Penicillin/Streptomycin at 6.5 ml per min. followed by 4–6 min perfusion using pre-warmed Dulbecco’s PBS containing 0.5 mg collagen per ml and 1 mM CaCl_2_ (pH7.4). The digested liver was transferred to Leibovitz-Buffer (Gibco, Life Technologies) and hepatocytes separated andcentrifuged at 50*g* for 5 min at 4 °C. The pellet was rinsed through a 40 μm cell and resuspended with 20 ml ice-cold hepatocyte wash media (Gibco, Life Technologies) containing 0.1 % Penicillin/Streptomycin. This was repeated seven times to yield highly purified hepatocytes. 80,000 hepatocytes per well were seeded using William's Medium E (10 % FKS, 0.025% Insulin (v/v), 0.1 % Glutamaxx (2mM) (Gibco, Life Technologies), 0.1% Penicillin/Streptomycin). After 6–8 h media was changed to remove non-attached hepatocytes.

### DY-635 and nanoparticle uptake by primary hepatocytes

Murine primary hepatocytes isolated and cultured on collagen-coated 96-well-plate for 2–3 days were used to measure the uptake of DY-635 (amino-terminated), DY-635[NP](NileRed) and [NP](NileRed) in absence and presence of cyclosporine A. Hepatocytes were washed with PBS before a 2 × inhibitor solution containing Krebs–Henseleit buffer (KHB, Biochrom AG), 2% foetal calf serum and 2 × cyclosporine A was added. This was then diluted 1:1 (v/v) with either 400 nM DY-635 (amino-terminated) or 10 μg NP in KHB. After 2 min (DY-635) or 30 min (NP) incubation at 37 or 4 °C media was removed and cells were washed twice with ice-cold PBS containing 50 μM cyclosporine A. Cells were lysed in 5 % acetonitrile and 0.1% Tween-20. Lysates were analysed on a Tecan plate reader (Tecan) (excitation: 488±09 nm, emission 530±20 nm). A NileRed-standard curve allowed calculation of intracellular NileRed-amounts.A correction factor was calculated from serial dilutions of the NP to correct for different NileRed concentration in [NP](NileRed) and DY-635[NP](NileRed). Data were normalised to cellular protein (Bradford).

### Polymer labeling

Conjugation of acid terminated PLGA (50:50) and amino-terminated DY-635 or DY-704 was performed *via* EDC chemistry (to label every hundredth polymer chain). 0.95 g COOH- terminated PLGA (Sigma-Aldrich, *M*_w_=7,000 to 17,000 g mol^−1^)) and 0.52 mg (2.72 μmol) EDC were reacted in 20 ml dried methylene chloride for 1 h at room temperature. Subsequently, 1 mg (1.36 μmol) NH_2_ terminated DY-635 or DY-704 (Dyomics GmbH, Jena, Germany), in 1 ml DMF was added and stirred at room temperature for 12 h. The solvent was evaporated under reduced pressure and the solid residue dissolved in acetone. The resulting PLGA-DY-635 or PLGA-DY-704 was precipitated (very fast) in cold water to generate a dispersion and this was further purified by dialysis against water using a Spectra/Por 3 dialysis membrane (Spectrumlabs, 3,500 g mol^−1^ cut-off). Finally, the product was filtered and lyophilised. The calculated labelling efficiency for conjugation was 77%. Size exclusion chromatography was performed on a Shimadzu system (Shimadzu) equipped with a SCL-10A system controller, an LC-10AD pump, an RID-10A RI detector, a UVD SPD-10AD UV/visible detector and a PSS SDV linear S, 5 μm column (8 × 300 mm) with chloroform/triethylamine/2-propanol (94:4:2) as eluent at 1 ml min^−1^, and the column oven was set to 40 °C. A calibration with low polydispersity polystyrene standards (ranging *M*_n_ from 380 to 128,000 g mol^−1^) was used.

### UV/Vis spectroscopy

UV/visible absorption spectra were recorded using a UV/visible spectrometer Specord 250 (Analytik Jena AG, Jena, Germany) in a 1 × 1 cm quartz sample cell. The labeling efficiency for conjugation was estimated via UV/visible spectroscopy using the molar extinction coefficient of the dyes and calculated via equation (1):


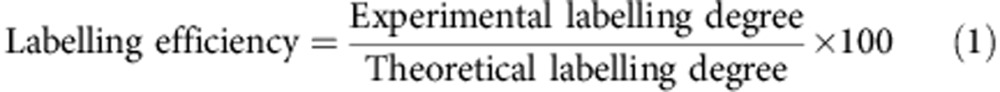


### Scanning electron microscopy (SEM)

NP suspensions were diluted with deionised water. One droplet was placed on a mica surface and lyophilised for 3 h. Finally, the samples were coated with platinum (5 nm), using a BAL-TEC MED020 sputtering device (Bal-Tec AG, Liechtenstein). SEM measurements were performed on a Zeiss (LEO) 1530 Gemini FESEM (Zeiss, Jena, Germany) operating at 8 to 10 kV using the InLens detector.

### Dynamic light scattering

Batch DLS was performed on a ZetaSizer Nano ZS (Malvern, Herrenberg, Germany) equipped with a He–Ne laser operating at a wavelength of 633 nm. Counts were detected at an angle of 173°. All measurements were conducted at 25 °C after an equilibration of 120 s in triplicate. For analyzing the autocorrelation function, the cumulative analysis and a NNLS algorithm (non-negative least-squares) were applied. Apparent hydrodynamic radii were calculated using to the Stokes–Einstein Equation (equation 2):


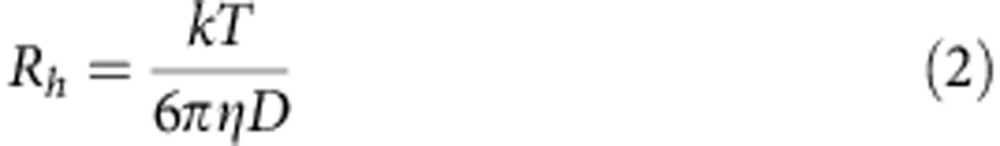


where, *R*_h_ = hydrodynamic radius, *k* = Boltzmann constant, *T* = absolute temperature, *η* = viscosity of the sample and *D* = apparent translational diffusion coefficient.

### Electrophoretic light scattering

Electrophoretic light scattering was used to measure the electrokinetic potential (zeta potential). Measurements were performed on a Zetasizer Nano ZS (Malvern Instruments) by applying laser Doppler velocimetry. For each measurement, 10–30 runs were carried out using the slow-field reversal and fast-field reversal modes at 150 V. Each experiment was performed in triplicate at 25 °C. The zeta potential (*ζ*) was calculated from the electrophoretic mobility (*μ*) according to the Henry Equation (equation 3) with *f(ka)* = 1.5:


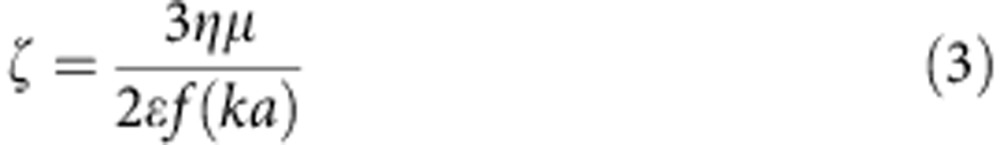


where *ε* = dielectric constant and *f(ka)* = Henry constant.

### Nanoparticle formulation

siRNA or indocyanine green (ICG) encapsulated in particles ([NP](siRNA), DY-635[NP](siRNA), [NP](ICG) or DY-635[NP](ICG)) were prepared by the double emulsion water/oil/water evaporation, as described^53^. siRNA encapsulation polyplexes of linear PEI (L-PEI) and siRNA (Supplementary Table 2) were prepared in pure water. L-PEI with a degree of polymerisation of 200 was synthesised as previously described^54^. L-PEI and siRNA were diluted in pure water to a stock concentration of 1 mg_ _ml^−1^. Polyplexes were formed of 8 μg siRNA and 9.8 μg L-PEI in a total volume of 250 μl, vortexed briefly, and incubated for 20 min at room temperature. A total mass of 100 mg PLGA or PLGA-DY-635 were diluted in 5 ml ethylacetate (20 mg ml^−1^). The polyplex solution was added and the mixture immediately sonicated for 20 s (VibraCell VC505 (500 W); Sonics) with a replaceable 1/8" tapered microtip, and 30% amplitude for 20 s. An aqueous solution of 3% (w/v) poly(vinyl alcohol) (PVA, M_W_ = 67,000 g mol^−1^, 86.7 to 88.7 mol% hydrolysed, Sigma Aldrich) (5 ml, PVA diluted in ddH_2_O) was then added to the first emulsion and sonicated as for the first emulsion. The double emulsion was added to 45 ml ddH_2_O. Ethylacetate evaporation was performed with stirring overnight. The particles were washed by centrifugation (14,000 *g*, 10 min) and twice resuspended in ddH_2_O. Particles were lyophilised and stored at – 20 °C until use. For encapsulation of DY-780 or Nile red, the polymer and dye were diluted in acetone to a final polymer concentration of 7.5 mg ml^−1^ with 2% (w/w) of each dye. The polymer dye solution was transferred to a syringe and automatically injected (13.02 ml h^−1^) into a three-fold excess of ultra-pure water. To stabilise the formed NPs and to enable lyophilisation 1 μg PVA was added to 100-mg polymer. Finally the NP solution was lyophilised and stored at −20 °C until use.

### Asymmetric flow field-flow fractionation

AF4 was performed on an AF2000 MT System (Postnova Analytics, Landsberg, Germany) coupled to an UV (PN3211, 260 nm (Postnova)), RI (PN3150), multi-angle light scattering (MALLS, PN3070, 633 nm (Postnova)) and DLS (ZetaSizer Nano ZS; Malvern) detector. The eluent was delivered by three different pumps (tip, focus, cross-flow) and the sample injected by autosampler (PN5300) into the channel. The channel has a trapezoidal geometry and an overall area of 31.6 cm^2^. The nominal height of the spacer was 500 μm. A regenerated cellulose membrane with a molar mass cut-off of 10 kDa served as accumulation wall. All experiments were carried out at 25 °C with pure water as eluent. An amount 10 μl of sample (1 mg ml^−1^) was injected with an injection flow rate of 0.2 ml min^−1^ and a cross-flow rate of 1.2 ml min^−1^ for 7 min (detector flow rate 0.5 ml min^−1^, focus flow rate 1.5 ml min^−1^). After the focusing step, the cross-flow rate was reduced under an exponential gradient (0.4) within 10 min to 0 ml min^−1^. The cross-flow was kept constant at 0 ml min^−1^ for 40 min to ensure complete elution. All measurements were in triplicate.

### Determination of siRNA content

siRNA content of the NPs was determined by determination of total phosphorus according to Chen *et al.*^55^ Freeze-dried NPs (10–16 mg) and phosphorus standards (0–0.195 μmol, KH_2_PO_4_, Sigma-Aldrich) were placed in separate tubes. After adding 0.45 ml of 8.9 N H_2_SO_4_, tubes were heated at 210 °C for 25 min. After cooling to room temperature, 150 μl H_2_O_2_ (30 wt. %) were added and samples were reheated at 210 °C for 30 min. After cooling 3.9 ml deionised water and 0.5 ml of ammonium molybdate (VI) tetrahydrate solution (2.5 wt. %) were added. After vortexing (20 s), 0.5 ml of L-ascorbic acid solution (10 wt. %) was added and the tubes vortexed again for 20 s. Tubes were capped and heated for 7 min at 80 °C. Absorbance of solutions was measured at 820 nm on a Specord 250 UV/visible—spectrometer (Analytik Jena AG) in a 1 cm quartz cell. siRNA content was determined based on phosphorous content and number of base pairs in the siRNA.

### Multispectral optoacoustic tomography

MSOT was performed as described^56^. Briefly, a commercial MSOT (iThera medical, Munich, Germany) with an array of 256 cylindrically focused transducers was disposed around the mouse and simultaneously collected signals from an optical plane. CD1 or athymic Nude-Foxn1^nu^ mice bearing a MDA-MB-231 human breast cancer xenograft were placed inside the tomograph. Multispectral image stacks were taken from the whole abdomen immediately before and 20 min after injection of 7 μg of either DY635-[NP](DY-780), [NP](ICG), DY-635[NP](ICG) or DY-704[NP](−) per g BW. DY-780 could be only detected by MSOT after release from the NPs. DY-780, DY-704 and ICG signals could be spatially localised upon spectral unmixing of MSOT images and comparison with previously recorded DY-780 spectra. Animals were frozen immediately after the imaging experiment for validation by multispectral epi-illumination cryoslice imaging^57^.

### Dynamic cell culture

HUVEC and HepaRG cells were co-cultured using a microfluidic-supported chip. (Supplementary Fig. 2). HepaRG cells were cultured as described^58^. 2.7 × 10^4^ cells per cm^2^ were seeded in William’s E medium supplemented with 10% foetal bovine serum, 100 U ml^−1^ penicillin, 100 g ml^−1^ streptomycin, 5 U ml^−1^ insulin and 5 × 10^−5^ M hydrocortisone-hemisuccinate and cultured for 2 weeks. For HepaRG differentiation, cells were cultured with 2% dimethylsulfoxide for two more weeks. Differentiated HepaRG cells were seeded at 4,000 cells per mm^2^ at the bottom of the chip cavities beneath the membrane. 300,000 HUVEC (4,300 cells per mm^2^) were seeded above the membrane and co-cultured with differentiated HepaRG cells in for 24 h before the uptake experiments. Cells were perfused with 50 μg ml^−1^ NPs in Williams E medium. Three independent experiments to assess DY-635[NP](−) uptake were done for each group (static and dynamic). For each time point three pictures were taken and 200 region of interest (ROI) per picture were analysed. Images in Fig. 6c were corrected by subtracting the mean local background intensity from the mean fluorescence intensity of a neighbouring ROI.

### Intravital microscopy

For IVM the jugular vein of male FVB/NRj mice (25 to 30 g BW) was cannulated and the liver exposed via a right lateral abdominal incision 70 pmol DY-635 g^−1^ BW (in dimethylsulfoxide (1 M) and diluted to 20 μM in 0.9% NaCl) or 7 μg DY-635[NP], DY-704[NP], DY-630[NP](NileRed) per g BW (1 mg ml^−1^ in 5% glucose) were injected i.v. IVM was performed using an inverted epifluorescence microscope (AxioObserverR Z1; Zeiss) as described previously^8^,^30^. DY-635 or DY-635[NP](−) were visualised at 633 nm (exposure time 40 ms). Co-localisation of DY-635 or DY-635(NP) and liver tissue was performed using 8-bit, grey-value tiff-images and ImageJ with a pseudo colour scale. For Fig. 7f pictures taken 8 min after injection of DY-635 and after 28 min (8 min after DY-635[NP](−) injection) were overlaid and colocalised pixels counted. This number was then corrected for the amount of colocalised pixel that might persist from the injection of free dye DY-635 using the following formula for DY-635 relative fluorescence decrease: FI(DY-635) [%] =71.6^−time/12.3^ + 151,9^−time/3.7^(Fig. 7c).

### *In vivo* confocal laser scanning microscopy

For *in vivo* confocal laser scanning microscopy a tail-vein catheter (30G) was placed in male FVB/NRj mice (25–30 g BW). The liver or/ and kidney was exposed and placed on a cover-slip. Images were acquired using a LSM-780 (Zeiss) using an air corrected × 40 plan-apochromatic objective (*NA*: 0.95). Lasers and filters used are summarised in Supplementary Table 3 NPs or free dyes were injected in similar concentration as described above via the tail vein-catheter.

### HMGCR-RNAi validation *in vitro*

Hepa1-6 cells were cultured in DMEM with 10% FBS and penicillin-streptomycin. 8 × 10^5^ cells per well were seeded in 12-well plates 24 h prior to transfection. Lyophilised DY-635[NP](siHMGCR) were dissolved in OptiMem (Life technologies) to 500 μg ml^−1^. Cells were washed twice in PBS and incubated with 1 ml DY-635[NP](siHMGCR). After 4 h media were changed to normal growth media for a further 12 or 20 h and *hmgcr* steady state transcript levels assessed by reverse transcriptase-quantitative PCR (RT-qPCR) (Supplementary Table 4). To measure HMGCR-protein knockdown efficacy, HEK293 cells were transfected with pRNAT H1.1 vectors encoding the *hmgcr*-shRNA used for NP encapsulations (Supplementary Table 3) as well as mouse GFP-HMGCR (419–887) instead of GFP (for plasmid and cloning information see Supplementary Fig. 3). After 24 h cells were lysed and protein levels analysed via anti-GFP immunoblotting^59^,^60^.

To confirm the heterologous *hmgcr* RNAi results by immunofluorescence analyses of endogenous HMGCR, anti-human HMGCR antibodies (Santa Cruz) were tested for recognition of purified recombinant HMGCR(aa419-887) fusion proteins of overexpressed murine GFP-HMGCR(aa419-887) and of endogenous HMGCR. For RNAi validations in HepG2-cells, cells transfected with GFP-coexpressing RNAi vectors were processed for fluorescence analysis using a Zeiss CellObserver with an apotome. 16 h after transfection Z-stacks were accumulated and integrated intensities of SUM-projections of the anti-HMGCR signal were measured in ImageJ. Two independent cellular assays with *n*>16 cells were conducted.

The effects of siHMGCR#2 on gene expression were assessed in 8 × 10^5^ Hepa1-6 cells incubated for 24 h with 2 ml full growth media (DMEM/F12, 10% FCS, 1% penicillin/streptomycin) at 37 °C, 5% CO_2_. Directly before transfection lyophilised DY-635[NP](siHMGCR) or DY-635[NP(siCtrl) were dissolved in OptiMEM (Life Technologies) to a final concentration of 100, 200 or 500 μg NP per ml. Growth Media were removed and the cells washed twice with PBS. 1 ml of the NP-solutions was added to the cells. Four hours later the NP was removed and full growth media added. Cells were harvested after an additional 14 or 20 h for RT-qPCR. See Supplementary Table 2 for primer-pairs. Results of three independent experiments with three technical replicates were used for *in vitro* gene expression analysis. Data are mean±s.e.m.

### *In vivo* RNAi

A venous access port was placed in male FCB/NRj mice at 5 weeks. The mice were treated with antibiotics for 7 days to prevent infection. Fourteen days after the operation lyophilised [NP](siHMGCR), DY-635[NP](siHMGCR) or DY-635[NP](siCtrl) were dissolved in 5% glucose to a concentration of 1 mg NP per ml. 7 μg NP (7 μl) per g BW was administered twice i.v. within 24 h. The mice were sacrificed 16 h after the second injection and blood collected into heparinised tubes. Plasma and organs were frozen for subsequent analysis. Metabolites were analysed from randomised, blinded samples of plasma (DRI-CHEM 3500i; Fujifilm). Gene expression was analysed in randomised tissue homogenates. *n*=4–9 (individual number are in the legend for Fig. 8).

### Statistics

Data were tested for normal distribution and analysed by appropriate analysis of variance (ANOVA) models as described in figure legends. *Post-hoc* analysis was performed using Dunnett’s test when comparing multiple groups with a single control or Tukey test when performing *post hoc* pair-wise analysis.

## Author contributions

Adrian T. Press: Planning, performing and analyzing experiments (Figs 1a–d, 2b,h, 5, 6, 7, 8a,d,e), Planning experiments (Figs 3, 4a, 5, 6), statistical analysis of the data, preparation of graphs and figures; wrote parts of the manuscript. Anja Traeger: Planning and supervising the particle formation and characterisation; wrote parts of the manuscript. Alexander Mosig: Planning, performing and analyzing experiments (Fig. 5a–d). Christian Pietsch: Planning, performing and analyzing the polymer-synthesis and characterisation (Supplementary Fig. 1a,c, 2d,f). Michael Wagner: Planning and performing the characterisation of the nanoparticles and loading efficiency (Fig. 2c, Supplementary Fig. 1). Mark Clemens: Planning and analyzing *in vivo* LSM experiments (Fig. 5b), planning and analyzing primary hepatocyte experiments (Figs 1d and 2h), planning statistical analysis, writing portions of the manuscript. Nayla Jbeily: Mainly contributed to the performance of intravital microscopy experiments. Nicole Koch: Planning, performing and analyzing experiments for RNAi and anti-HMGCR antibody (Fig. 7b,c and Supplementary Fig. 3). Michael Gottschaldt: Scientific supervision of nanoparticle analysis, supervised and designed nanoparticle characterisation. Volodymyr Ermolayev and Nicolas Bézière: Performed MSOT experiments. Vasilis Ntziachristos: Planned, designed and supervised MSOT analyses. Jürgen Popp: Suggested use of MSOT to image nanoparticles; planned and designed biophotonic analyses. Michael M. Kessels: Planning and scientific supervision of *hmgcr* cloning and immunodetections, wrote parts of the manuscript. Britta Qualmann: Planning and scientific supervision of *hmgcr* cloning and immunodetections, wrote parts of the manuscript. Ulrich S. Schubert: Design of study (chemistry), planning and supervision of nanoparticle design, wrote the manuscript. Michael Bauer: Design of study (life sciences), planning of functionalised nanoparticles, wrote the manuscript.

## Additional information

**How to cite this article:** Press, A. T. *et al.* Cell type-specific delivery of short interfering RNAs by dye-functionalised theranostic nanoparticles. *Nat. Commun.* 5:5565 doi: 10.1038/ncomms6565 (2014).

## Supplementary Material

Supplementary Information Supplementary Figures 1-3 and Supplementary Tables 1-4.

## Figures and Tables

**Table 1:** Characterisation of nanoparticles.

	**Targeting moiety**	**Active payload**	Size* **(d in nm)**	**Surface charge**† **(in mV)**	**Further data**
[NP](−)	−	—	210±50	−7±2	
[NP](siRNA)	−	siRNA‡	190±32	+78±5	
[NP](NileRed)	−	NileRed	196±24	−25±18	
[NP](ICG)	−	ICG	145±45	−24±6	
DY-635[NP](−)	+	—	170±5	−13±5	
DY-635[NP](siRNA)	+	siRNA‡	176±22	−11±6	0.78§, 87.4±2.5%∥
DY-635[NP](NileRed)	+	NileRed	142±18	−19±7	
DY-635[NP](ICG)	+	ICG	151±32	−63±3	
DY-704[NP](−)	+	—	155±37	−42±6	

^*^Determined by DLS.

^†^Zeta potential.

^‡^Complexed with LMW PEI, N/P10.

^§^Rg/Rh determined by AF4.

^∥^Encapsulation efficiency.
