# Supplementary material for: Cell type-specific delivery of short interfering RNAs by dye-functionalised theranostic nanoparticles
Source: Nat Commun. 2014 Dec 3;5:5565. doi: 10.1038/ncomms6565 (PMC4268698; doi:10.1038/ncomms6565)
Supplement: Supplementary Information — Supplementary Figures 1-3 and Supplementary Tables 1-4. [file ncomms6565-s1.pdf]

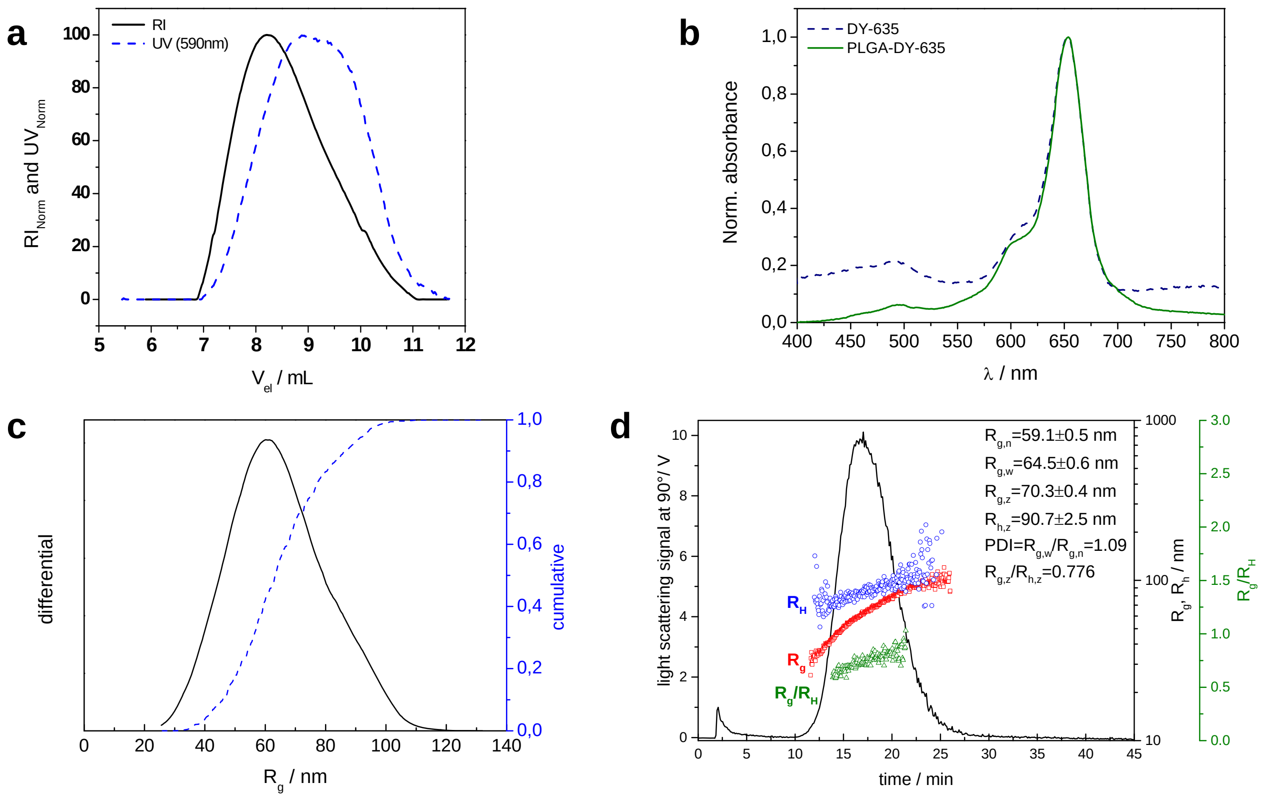

Supplementary Figure 1: Characterization of polymers and nanoparticles. (a) PLGA is labeled with DY-635 *via* 1-ethyl-3-(3-dimethylaminopropyl)carbodiimide (EDC) coupling. Successful coupling leads to a strong UV-Vis response in the SEC trace at 590 nm (dotted blue line). . **(b)** UV/Vis spectra of DY-635 and PLGA-DY-635 showing λ_{max} at 653 nm. **(c)** The AF4 based R_g distribution of DY-635[NP](-) show low polydispersity and uniformity of DY-635[NP](-). **(d)** AF4 measurements show a monomodal distribution (polydispersity index of 1.09) of spheric DY-635[NP](-). $R_{g,i}$ represent the different averages of the root-mean-square radius. The hydrodynamic radius, R_h, represents the diffusion based radius. Furthermore, the calculated shape factor (R_g/R_h) indicates a spherical geometry of the analyte.

a

b

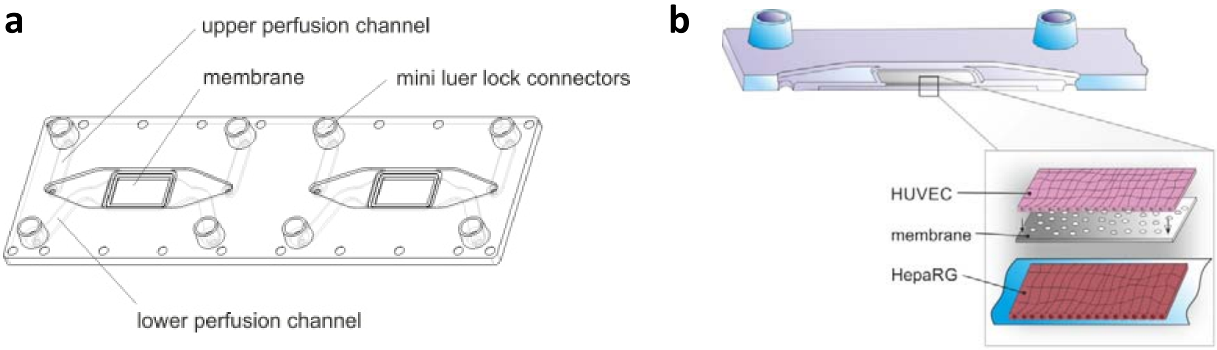

Supplementary Figure 2: Microfluidics-supported chip for coculture of HepaRG with HUVEC cells under flow conditions ("organoid").

(a) Structure of a microfluidic-supported chip, **(b)** scheme of the co-culture of HepaRG and HUVEC. Cells are separated by a porous membrane (pore diameter of 100 µm). NP were perfused exclusively via the upper perfusion channel with direct contact to the HUVEC cells to mimic anatomy of liver sinosods regarding the architecture of endothelium, Disse space and hepatocytes.

a

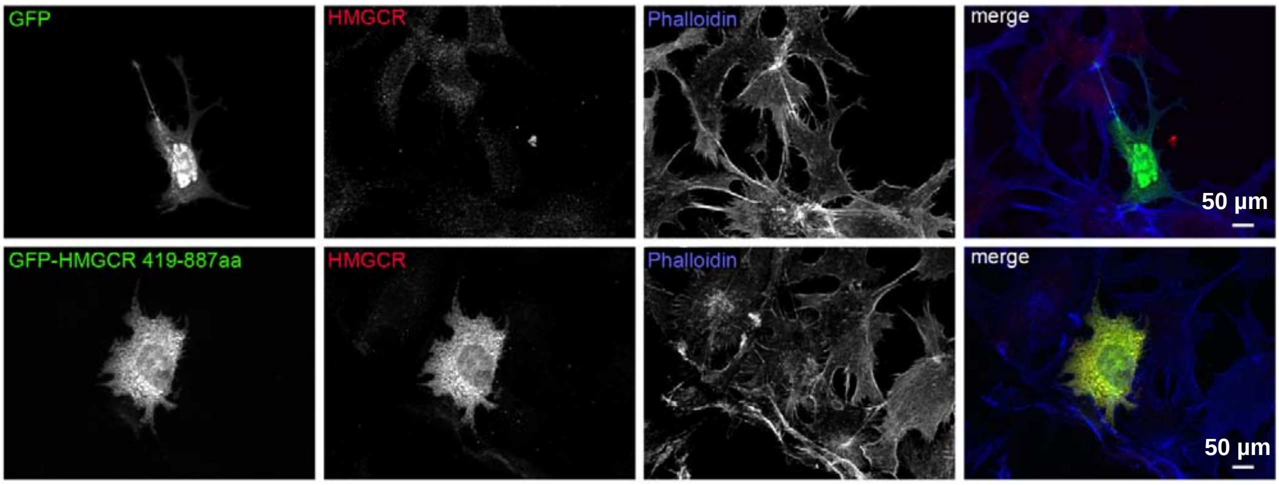

b

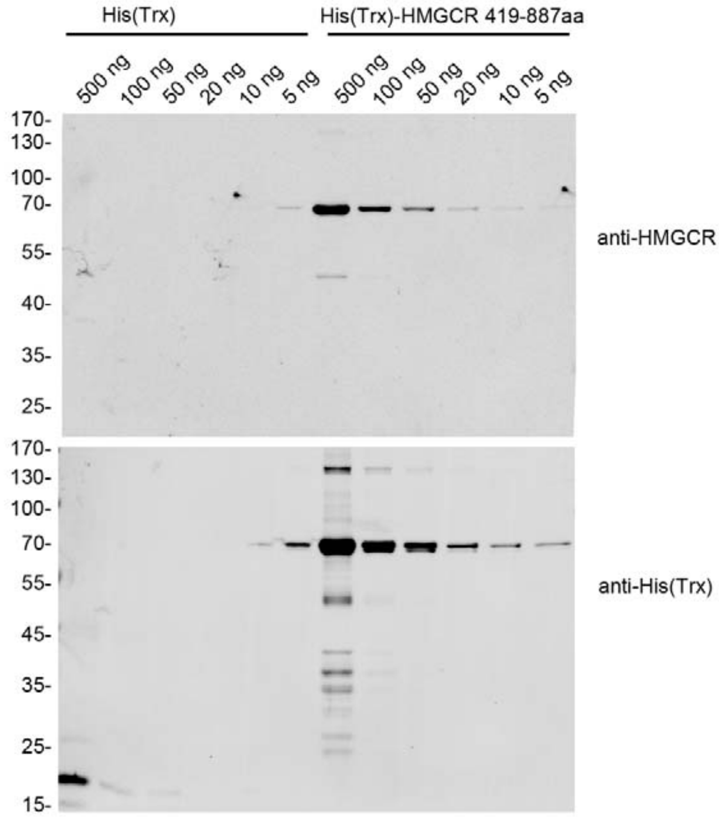

Supplementary Figure 3:
Anti-HMGCR characterization.
(a) Antibody validation in HepG2 cells overexpressing GFP-HMGCR 419-877 or GFP as control. The anti-HMGCR antibody specifically recognizes GFP-HMGCR 419-877 and shows a weak endogenous HMGCR signal. Cells were additionally stained with Phalloidin to visualize untransfected cells. **(b)** Antibody validation via Western Blot. Distinct, different amounts of recombinant His(Trx) and His(Trx)-HMGCR fusion protein were blotted and detected with anti-HMGCR and anti-His(Trx) antibodies, respectively. The anti-HMGCR antibody detects specific bands within 500 ng to 10 ng HMGCR fusion protein.

Supplementary Table 1 List of dyes and tissue specific combinations

Dye[1]	Sulfonic residues	Suggested polymers[2]	Target tissue	Optical modality[3]
**DY-680 amine**	1		Hepatocyte	LSM, NIR-Imaging, MSOT, Epi
**DY-780 amine**	1		Hepatocyte	NIR-Imaging, MSOT, Epi
**DY-880 amine**	1		Hepatocyte	MSOT
**DY-635 amine**	1		Hepatocyte	LSM, NIR-Imaging, Epi
**DY-735 amine**	1		Hepatocyte	LMS, NIR-Imaging, MSOT, Epi
**DY-835 amine**	1	**Poly(D,L-lactide-*co*-glycolide), acid terminated, 50:50** (CAS Number 26780-50-7)<br>**Poly(D,L-lactide) , acid terminated, 50:50** (CAS Number 26680-10-4)<br>**Polycaprolactone** (CAS Number 24980-41-4)	Hepatocyte	NIR-Imaging, MSOT, Epi
**DY-730 amine**	1		Hepatocyte	LSM, NIR-Imaging, MSOT, Epi
**DY-830 amine**	1		Hepatocyte	NIR-Imaging, MSOT, Epi
**DY-750 amine**	1		Hepatocyte	NIR-Imaging, MSOT, Epi
**DY-850 amine**	1		Hepatocyte	NIR-Imaging, MSOT, Epi
**ICG NHS**	2		Hepatocyte	NIR-Imaging, MSOT, Epi
**DY-778 amine**	4		Renal parenchymal cells	NIR-Imaging, MSOT, EPI<br>NIR-Imaging, MSOT, EPI
**DY-878 amine**	4		Renal parenchymal cells	NIR-Imaging, MSOT, EPI
**DY-704 amine**	3		Renal parenchymal cells	MSOT
**DY-754 amine**	4		Renal parenchymal cells	NIR-Imaging, MSOT, Epi
**DY-854 amine**	4		Renal parenchymal cells	NIR-Imaging, MSOT, Epi
**IRDye800CW NHS**	4		Renal parenchymal cells	NIR-Imaging, MSOT, Epi

[1] Amine-terminated dyes are provided by Dyomics GmbH, Germany, IRDye80 CW was purchased from Li-Cor as NHS-ester, ICG-NHS ester from Intrace medical .

[2] Polymers are purchased from Sigma Aldrich.

[3] LSM: (confocal) Laser scanning microscopy (also in vivo), may need a tuneable Ti:sapphire laser to excite NIR-Dyes; NIR-Imaging: devices such as IVIS or Maestro (PerkinElmer), MSOT: Multi-spectral optoacustic tomograph, e.g. iTheraMedical GmbH, EPI: Epifluorescence Microscope equipped with suitable filter-sets and proper (N)IR-Sensitive detection systems.

Note that we only can provide a small selection of dye-polymer-pairs since noumerous dye-functionalization and polymers are avaivable wich can lead to succsessful coupled functional polymers.

Supplementary Table 2 siRNA sequences used for hmgcr-RNAi

Name	Target Ref. Seq	Primer Sequence (5' → 3')	
		sense	antisense
**RNA#1**	NM_008255.2	acuugcucaauguccau	gcauggacauugagcaagu
**RNA#2**	NM_008255.2	guaccugccuuacagag	aucucuguaaggcaggua

Supplementary Table 3 Excitation and emission wavelength used for in vivo confocal scanning microscopy

Confocal laser scanning microscopy	Excitation (nm)	Emission (nm)	Strucutre
**NAD(P)H autofluorescence**	405±30	410-440	liver architecture
**NileRed**	488	530-630	payload
**DY-635/ DY-704**	633	LP 640	targeting moiety

Supplementary Table 4 Primer sequences used in qPCR

**Gene name**	**Host**	**Target Ref. Seq**	**Primer Sequence (5' → 3')**	
			forward	reverse
hmgcr	Mouse	NM_008255.2	tgg ttc ttt ccg tgc tgt gt	acc agt ttc cag ctt gtg gt
hprt	Mouse	NM_013556.2	tga cac tgg caa aac aat gca	ggt cct ttt cac cag caa gct
